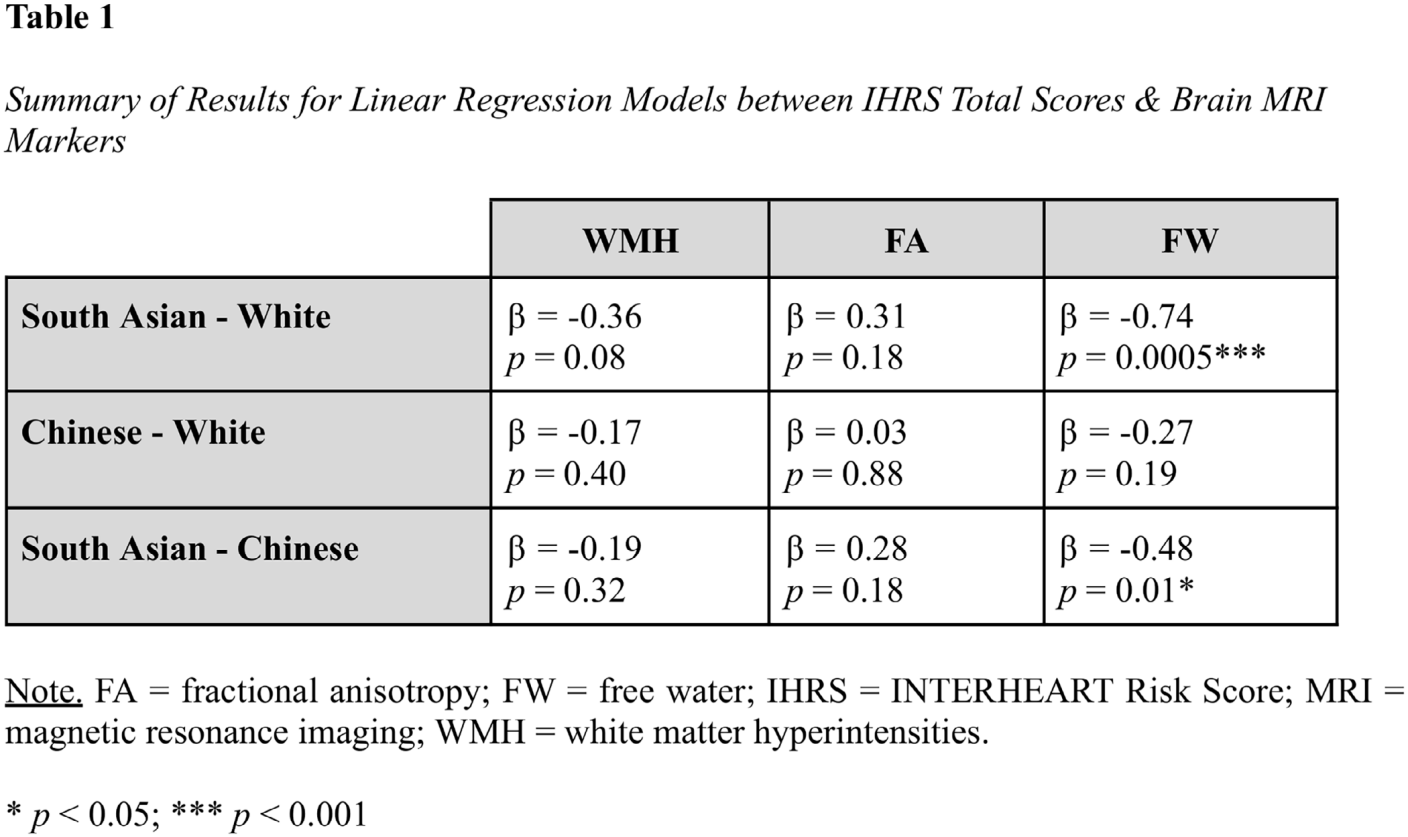# Ethnic Differences in the Association between Vascular Risk and Brain MRI Markers of Dementia: Findings from the CAMERA Study

**DOI:** 10.1002/alz70861_108971

**Published:** 2025-12-23

**Authors:** Rohina Kumar, Katie L Vandeloo, Simran Malhotra, Angelina Zhang, Sarah‐Mei Chen, Rachel Yep, Tulip Marawi, Harleen Rai, Alexander J Nyman, Georgia Gopinath, Madeline Wood Alexander, Silina Z Boshmaf, Walter Swardfager, Sandra E. Black, Maged Goubran, Jennifer S Rabin

**Affiliations:** ^1^ Hurvitz Brain Sciences Program, Sunnybrook Research Institute, Toronto, ON Canada; ^2^ Department of Pharmacology and Toxicology, University of Toronto, Toronto, ON Canada; ^3^ Division of Neurology, Department of Medicine, University of Toronto, Toronto, ON Canada; ^4^ Rehabilitation Sciences Institute, University of Toronto, Toronto, ON Canada; ^5^ Department of Medical Biophysics, University of Toronto, Toronto, ON Canada; ^6^ Harquail Centre for Neuromodulation, Sunnybrook Research Institute, Toronto, ON Canada

## Abstract

**Background:**

Vascular contributions to Alzheimer’s disease and related dementias (ADRD) are well established. Interestingly, South Asian individuals tend to have a higher vascular burden compared to White individuals, while Chinese individuals generally exhibit a lower vascular burden. However, epidemiological data on dementia incidence among Asian populations are limited and often contradictory. Some studies report a higher incidence of dementia in South Asians compared to Chinese and White individuals, whereas others suggest a lower risk in both South Asians and Chinese populations relative to White individuals. This study investigated whether ethnicity moderates the association between vascular risk factors and brain magnetic resonance imaging (MRI) markers of ADRD among South Asian, Chinese, and White adults.

**Method:**

Participants included 113 cognitively unimpaired adults (mean age = 67.1 ± 6.6 years, 70.8% female) of South Asian (*n*=36), Chinese (*n*=39), and White (*n*=38) descent, aged 55‐85, from the *Canadian Multi‐Ethnic Research on Aging (CAMERA)* study. Vascular risk burden was assessed using the INTERHEART Risk Score (IHRS). Brain imaging markers included log‐transformed white matter hyperintensities (WMH) derived from FreeSurfer software (v.8), as well as whole‐atlas fractional anisotropy (FA) and free water (FW) diffusion obtained using UKF tractography‐based analyses. Linear regression models tested for interactions between IHRS and ethnicity on WMH, FA, and FW. We adjusted for age, sex, years of education, income, intracranial volume, and white matter hyperintensities where relevant.

**Result:**

Ethnicity moderated the associations between IHRS and brain markers (Table 1). Higher IHRS was associated with greater WMH burden, lower FA, and higher FW in White participants. Chinese participants exhibited a similar pattern to White individuals. South Asian participants showed a counterintuitive pattern, where higher IHRS scores were associated with lower WMH burden, higher FA, and lower FW.

**Conclusion:**

These findings suggest that the impact of vascular burden on brain aging may differ across ethnic groups. However, the small sample size and preliminary nature of the data warrant cautious interpretation. Further research with larger samples is needed to replicate these findings, clarify the underlying mechanisms, and inform equitable healthcare strategies.